# Left Atrial Myxoma With Aortic Insufficiency Leading to Cerebrovascular Accident

**DOI:** 10.7759/cureus.39048

**Published:** 2023-05-15

**Authors:** Kevyn Niu, Manuel Torres Velez, Yizhi Lin

**Affiliations:** 1 Internal Medicine, Blake Medical Center, Bradenton, USA

**Keywords:** bicuspid aorta, adult cardiac surgery, aortic valve insufficiency, myxoma, right atrial myxoma

## Abstract

Primary cardiac tumors are extremely rare and can lead to significant neurologic symptoms if not diagnosed and treated appropriately. Cardiac myxomas represent the most common subtype of cardiac tumors and are typically located on the left side of the heart and, when diagnosed appropriately with echocardiography, are typically treated with surgical excision. Simultaneous presentation of myxoma and valvular insufficiency is rare and under-documented. This is a rare case of a patient with a left atrial myxoma and aortic insufficiency leading to cerebrovascular symptoms.

## Introduction

Cardiac myxomas are the most common type of primary intracardiac tumors and are typically benign. Despite its relative frequency, cardiac myxomas are typically only found in 0.5-1 per 1,000,000 individuals in the general population, making the diagnosis a challenge in clinical practice [1]. The most common sequela of cardiac myxomas is thromboembolism to the brain due to the hypercoagulable property of these mobile lesions, causing nonspecific cerebrovascular accident symptoms. Most myxomas are found in patients between the age of 30 and 60 [2], an age group in which valvular abnormalities are rare. After an extensive literature review and to the best of our knowledge, there has been minimal research on patients with both an intracardiac myxoma and aortic valvular insufficiency. We present a rare case of a patient with a cardiac myxoma and valvular insufficiency leading to neurological impairment, with eventual surgical resection and full recovery.

## Case presentation

We present a case of a 42-year-old male with a past medical history of migraines, hyperlipidemia, and bicuspid aorta who presents to the hospital with a chief complaint of sudden right upper extremity numbness. Our patient reports sudden right upper extremity numbness two days prior to admission, followed by an episode of migraine with a typical aura. One previous episode of this phenomenon was noted three months ago involving the right leg, lasting 30-40 minutes, and resolving spontaneously. No weakness, paresthesia, or pain was noted. Symptoms quickly resolved, and the patient was asymptomatic at presentation. Physical examination at this time was unremarkable. Vital signs were stable.

**Figure 1:**
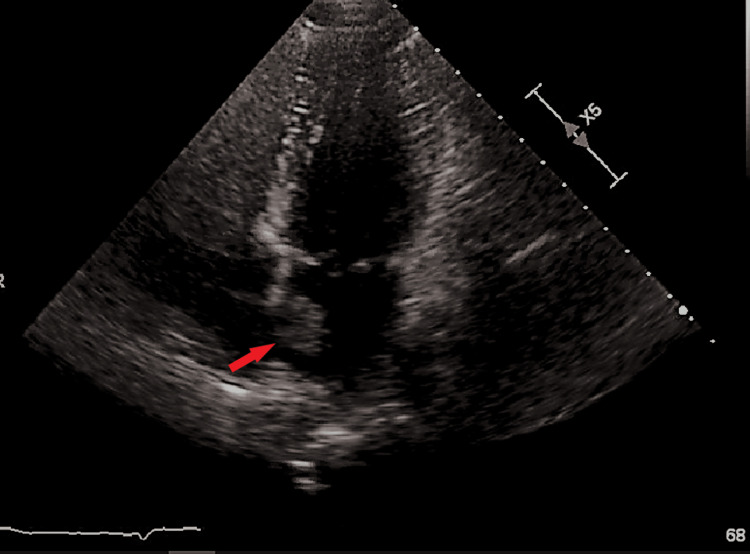
TEE, apical four-chamber view. The red arrow demonstrates a mobile mass on the left atrium, approximately 13 x 15 mm, with mild aortic insufficiency

EKG demonstrated sinus rhythm. CT imaging completed in the emergency department revealed a 6 mm hypodensity in the left periventricular white matter adjacent to the lateral ventricles superior portion of the basal ganglia. MRI brain with contrast demonstrated additional small foci of subacute non-hemorrhagic infarction in the distribution of the left middle cerebra artery and posterior cerebral artery, concerning cardioembolic sources. Subsequent transthoracic echocardiogram completed revealed an ejection fraction of 65-70%, no regional wall abnormalities, and a bicuspid aortic valve with mild/moderate regurgitation, along with a mobile mass noted on the left atrium (Figure 1). Transesophageal echocardiogram (TEE) with bubble study revealed a definite spherical mobile mass measuring 15 x 13 mm on the left side of the interatrial septum. The remainder of the findings on TEE were unremarkable. A left heart catheterization revealed no significant coronary artery disease. 2+ aortic insufficiency was noted at this time on TEE.

Cardiothoracic surgery was consulted, and the patient underwent open heart surgery with resection of the left atrial mass. The postoperative course was unremarkable, and our patient was discharged on postoperative day three. Pathological examination post-discharge with immunohistochemistry revealed calretinin-positive, S100 focal-positive cells, likely of a benign cardiac myxoma, with no evidence of atypia, mitosis, or necrosis. Our patient was discharged without complications, with instructions to follow with their cardiologist following discharge.

## Discussion

Atrial myxomas are the most common benign cardiac tumor and are considered exceedingly rare, with an incidence of < 0.5%. Cardiac myxomas represent approximately 50% of primary cardiac tumors and are typically found within the left atrium, followed by fibroelastomas and lipomas [3]. The distribution of cardiac myxomas typically varies, with 75% being found in the left atrium, 15-20% in the right atrium, and the remainder in the ventricles. Embolization is more frequent with cardiac myxomas as a result of the preponderance of left-sided tumors. Neurological symptoms are the most common and are usually caused by surface thrombus embolization; much more rarely they are caused by fragmentation of the tumor; as such, consideration of anticoagulation therapy is warranted, as discussed below [4]. The majority of these symptoms are reversible, as seen in our patient [5]. Chest pain, palpitations, and dyspnea are also common presenting symptoms [6], secondary to cardiac outflow obstructive pathology. Patients can also present with constitutional symptoms due to interleukin overproduction [7]. Our patient reported no episodes of shortness of breath or dyspnea; this was likely due to the positioning of the myxoma on the interatrial septum, which would demonstrate less occlusive symptomology.

There have been few instances of atrial myxoma with aortic insufficiency recorded in the literature; our patient represents a very narrow patient population with both these conditions. The extant literature suggests that the distortion of the aortic root may be due to the movement of the myxoma during diastole [8]. Further investigation may be required to investigate correlations, if any exist, between aortic valvular abnormalities and left atrial myxomas.

Myxomatous tumors as described in our case are typically excised through open-heart surgery under cardiopulmonary bypass. Discussion regarding anticoagulation in the intraoperative period is warranted. Large embolic strokes may convert to hemorrhagic strokes, particularly in the first week after presentation [9]. Thus, the dose of anticoagulation required for open-heart surgery may trigger hemorrhagic transformation. There are no current guidelines regarding the titration of anticoagulation in the peri- and postoperative periods. Certain authors recommend the bridging of anticoagulation with a delay of open-heart surgery [10]. It is also worth mentioning that in certain populations where surgical intervention is contraindicated due to age or other mitigating factors, anticoagulation and thrombolytic therapy are viable primary therapies, typically with low-molecular-weight heparin or recombinant tissue plasminogen activator [11]. Our patient was able to tolerate surgical intervention well, with no pre-operative contraindications.

Long-term follow-up is important in cases of patients with intracardiac myxomas. The most common postoperative complication is atrial fibrillation [9,10]. Sahin et al. suggest that cardiac arrhythmias may be a result of atrial overload and secondary dilation of the atrial cavity [10]. The recurrence rate of myxoma is 2-3%, with increases of recurrence up to 23% in patients with Carney complex, a set of autosomal dominant conditions comprising myxomas of the skin and heart, lentiginosis, and endocrine abnormalities, typically caused by mutations in the tumor suppressor gene PRKAR1A on chromosome 17 [12]. Long-term monitoring with biannual transthoracic echocardiography has been suggested for monitoring; however, recent studies have questioned the benefits of frequent echocardiography in the setting of infrequent recurrence [13].

## Conclusions

Cardiogenic thromboembolisms should always be considered in young patients with cerebrovascular symptoms suggestive of stroke. Clots formed on the myxoma surface can detach and enter the bloodstream as emboli. Subsequent embolic vascular obstruction can lead to ischemic stroke. The first-choice treatment should be surgical, with anticoagulation therapy as a second-line therapy for those who cannot tolerate surgical intervention. More research into possible causative pathology between the development of intracardiac tumors and valvular insufficiency is warranted.
